# Genotyping Rotavirus RNA from Archived Rotavirus-Positive Rapid Test Strips

**DOI:** 10.3201/eid1701.101132

**Published:** 2011-01

**Authors:** Lester M. Shulman, Ilana Silberstein, Jacqueline Alfandari, Ella Mendelson

**Affiliations:** Author affiliations: Central Virology Laboratory, Tel Hashomer, Israel (L.M. Shulman, I. Silberstein, J. Alfandari, E. Mendelson);; Tel Aviv University, Ramat Aviv, Israel (L.M. Shulman, E. Mendelson)

**Keywords:** Rotavirus, genotyping, affinity purification, viruses, Israel, research

## Abstract

Genotyping circulating rotaviruses before and after introduction of rotavirus vaccine is useful for evaluating vaccine-associated changes in genotype distribution. We determined frequency of rotavirus genotypes among 61 rotavirus-positive children hospitalized in Israel during the 2005–06 rotavirus season. Accurate molecular epidemiologic data were recovered from affinity-concentrated rotavirus immobilized in rotavirus-positive bands from air-dried, diagnostic rotavirus rapid test strips (dipstick) stored at room temperature from 1 week to 5 years. G genotypes were identical for 21 paired dipsticks and suspensions, whereas dipsticks or suspensions detected an additional G genotype in 2 samples. RNA sequences from 7 pairs were identical. Phylogenetic analysis suggested previously unreported G2 sublineages and G9 lineages. The ease with which dipsticks can be stored at local facilities and transported to central reference laboratories can reverse increasing difficulties in obtaining geographically representative stool samples and expand surveillance to regions lacking adequate laboratory facilities.

Rotavirus infection is a leading cause of gastroenteritis in children <5 years of age worldwide; children with severe dehydration and electrolyte imbalance require hospitalization (*1*). In 2007 and 2008, the annual economic cost for hospitalization of rotavirus case-patients in Israel was US $4,578,489 in direct costs to the health system plus an additional household cost of $3,101,955 (*2*). Poor hygienic conditions and lack of appropriate medical facilities in developing countries result in high infant mortality rates (*1**,**3**–**5*).

The genes encoding the outer capsid viral proteins VP7 and VP4 form the basis of classification of group A rotaviruses into G and P genotypes, respectively (*6**,**7*). Two live oral vaccines, monovalent Rotarix vaccine (GlaxoSmithKline, Research Triangle Park, NC, USA) and pentavalent Rotatek vaccine (Merck, Rahway, NJ, USA) effectively reduced hospitalizations for subsequent infections with G1P[8], G2P[4], G3P[8], and G4P[8] rotaviruses by >85% (*8**,**9*). Rotavirus vaccines that are scheduled for inclusion in Israel’s national vaccination program after 2010 may selectively change relative distributions of naturally cocirculating rotaviruses of different G and P genotypes. According to analysis of voluntary immunizations before universal vaccination, the effectiveness of rotavirus vaccine in preventing rotavirus gastroenteritis–associated hospitalizations in Israel is already evident (*10*).

Simple to use, inexpensive, diagnostic rotavirus rapid test strips (dipsticks) identify group A rotavirus–positive stools in <30 minutes by lateral flow immunochromatography without indicating the genotype. Dipsticks are dipped into saline stool suspensions. If rotavirus is present, a colored band appears when indicator-linked rotavirus antibodies bind to the virus, and this complex is trapped on a band of membrane-bound rotavirus antibody on the dipstick. With their growing use at point-of-care facilities and in local hospital laboratories, fewer samples are available for genotyping by centralized reference laboratories. Obtaining geographically representative stool samples and expanding surveillance to regions lacking adequate laboratory facilities is becoming increasingly difficult. The ease with which dipsticks can be stored at local facilities and transported to central reference laboratories can help overcome these difficulties. We hypothesized that rotavirus trapped on rotavirus-specific antibody bands, equivalent to affinity concentrated rotavirus, might yield genotyping-quality RNA from dipsticks that are easily archived locally and transportable to centralized laboratories. In this study, our main objective was to determine the feasibility of recovering molecular epidemiologic data from rotavirus immobilized on air-dried diagnostic rotavirus dipsticks to compensate for the decreasing number of fecal samples reaching central reference laboratories.

## Methods

Stool samples from 311 children hospitalized with acute gastroenteritis at Sheba Medical Center, Tel Hashomer, Israel, during May 15, 2005–May 15, 2006, were suspended in saline. Rotavirus dipsticks (Hy Laboratories LTD, Rehovot, Israel) were used to identify rotavirus-positive stool samples. (For comparison with rotavirus ELISA with Dako reagents [Dako Diagnostics LTD, Glostrup, Denmark], diagnostic dipsticks from Hy-Laboratories and Novamed [Novamed, Jerusalem, Israel] had sensitivities of 98.5% ± 3% and 92.3%, and specificities of 92.5% ± 1% and 93.5%, respectively [L.M. Shulman et al., unpub data].) The section of the dipstick containing the rotavirus-specific bands from 14 rotavirus-positive dipsticks that had been stored at room temperature for at least 1 week were excised and placed into 120 µL saline.

Rotaviral RNA was extracted from these rotavirus-specific bands and from the corresponding saline stool suspensions by using QIAmp Viral RNA Mini kits (QIAGEN, Valencia, CA, USA). RNA amplified from rotavirus-specific bands from dipsticks archived at room temperature 5 years earlier was similarly extracted. RNA was amplified by using generic primers for reverse transcription–PCR (RT-PCR) (QIAGEN OneStep RT-PCR kits, QIAGEN) followed by heminested PCR (AmpliTaq Gold, Applied Biosystems, Foster City, CA, USA) with genotype-specific primers to identify G and P genotypes as described by Gouvea et al. (*11*) and Gunasena et al. (*12*), respectively. RT-PCR products were gel purified (QIAQuick Gel Extraction Kit, QIAGEN) or purified directly (HiPure PCR Product Purification Kit; Roche Applied Science, Indianapolis, IN, USA) from the PCR mix and sequenced by using an ABI PRISM Dye Deoxy Terminator cycle sequencing kit (Applied Biosystems) and the genotype-specific primer and common primer from the heminested PCR reaction described. Reaction mixtures were analyzed on Applied Biosystems model 373 DNA automatic sequencing system to confirm the RT-PCR genotyping results and for phylogenetic analysis.

Sequences of the same genotype were truncated to the longest common length by using the Sequencher program (Genecodes, Ann Arbor, Michigan, USA). Nearest-neighbor phylogenetic trees were constructed from these sequences after the data were bootstrapped 1,000 times with Clustal X (*13*). The tree was analyzed by using NJplot (*14*). Sequences have been deposited in European Molecular Biology Laboratory/GenBank/DNA Data Bank of Japan under accession nos. FN298858–FN298875, FN582119–FN582124, and HQ174462–HQ174463. (Isolate names are linked to their specific accession numbers in Table A1).

This study was approved by the institutional review board of Chaim Sheba Medical Center (SMC-7606-09). Experiments were not performed on humans. All personal identification was removed from the remnants of fecal samples sent to the National Center for Viral Gastroenteritis for rotavirus analysis. The viral RNA used for this study was obtained from these anonymous samples.

## Results

To assess the suitability of RNA extracted from dipsticks for determining the VP7 genotype, we first determined the VP7 G genotype of rotavirus in rotavirus-positive clinical samples from children hospitalized during May 15, 2005–May 15, 2006. Of 311 stool samples, 61 were determined to be rotavirus positive by using diagnostic rotavirus dipsticks. The G genotype and P genotype of RNA in 54 of the 61 samples was determined by the size of the heminested PCR amplification product as described. Mixed infections, e.g., >1 G (6 samples) and P genotypes (2 samples) in the same fecal suspension, were found in 8 (15%) of the 54 positive samples. Taking into account mixed infections, we found G1, G2, G3, and G9 G genotypes in 79%, 5%, 2%, and 15%, respectively, of the 62 rotaviruses identified in the 54 clinical samples. The frequencies of associated G and P genotypes for isolates from the 2005–2006 rotavirus season were 3% G1P[4], 76% G1P[8], 5% G2P[4], 2% G3P[8], 3% G9P[4], and 11% G9P[8].

We chose 18 rotavirus-positive clinical samples with which to study the feasibility of recovering molecular information from dipsticks. Thirteen were from children infected with 1 G genotype of rotavirus and 5 from children simultaneously infected with rotaviruses belonging to 2 different G genotypes. Of the 13 infections by a single G genotype, 8 were G1, 3 were G2, and 1 each were G3 and G9. All 5 mixed infections, RoV-24_ISR05, RoV_41_ISR05, RoV_56_ISR06, RoV_57_ISR06, and RoV_60_ISR06, were simultaneous infections with G1 and G9 genotypes. Mixed infections were identified by specific product size on gels and confirmed by partial sequencing of the G1 product for all 4 patients whose G9 sequences appear in the Figure.

**Figure:**
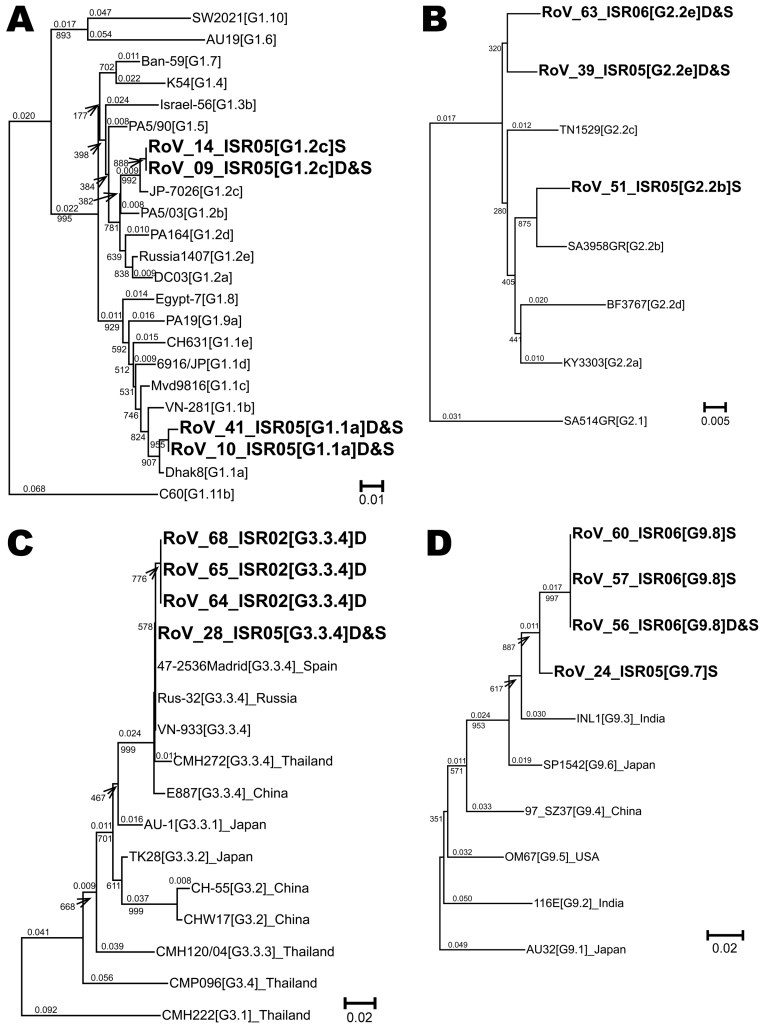
Neighbor-joining phylogenetic trees for viral protein (VP) 7 G1, G2, G3, and G9 genotypes of hospitalized children in Israel, including sequences recovered from archived rotavirus dipsticks. Representative isolates for lineages and sublineages of VP7 genotypes G1 (A), G2 (B), G3 (C), and G9 (D) were chosen from Phan et al. (15) Page and Steele (16), Wang et al. (17), and Martinez-Laso et al. (18), respectively, and the sequences were downloaded from the European Molecular Biology Laboratory/GenBank/DNA Data Bank of Japan. These sequences were aligned with Israeli sequences by using the Sequencher program (Genecodes, Ann Arbor, MI, USA) and truncated to the longest segment common to all sequences in the alignment; 515 nt for G1, 547 nt for G2, 274 nt for G3, and 207 nt for G9. Each of the 4 phylogenetic trees was prepared by using ClustalX (13) for data bootstrapped 1,000× and was analyzed with NJplot (14). Whole numbers indicate bootstrap values for branches; fractional numbers indicate genetic distances. The genotype, lineage, and, where relevant, sublineage of each isolate appears in brackets after the name of the isolate: for example, KY3303[G2.2a] is VP7 genotype G 2, lineage 2, sublineage a for isolate KY3303. A letter at the end of the name of the Israeli sequences indicates the source of the RNA (D for dipstick or S for fecal suspension). D&S appears when the sequences were identical. The GenBank accession numbers for all sequences in this figure appear in Table A1. Scale bars indicate percent of nucleotide substitutions per site.

RNA was extracted from rotavirus-positive bands of dipsticks from the 18 clinical samples. The dipsticks had been stored for >1 week at room temperature. The quality of the RNA from all 18 extractions was sufficient to enable determination of VP7 G genotypes by heminested RT-PCR. The genotypes obtained from the RNA extracted from dipsticks were identical to those from RNA extracted from corresponding saline suspensions for 14 of the 18 rotavirus-positive stool samples, including 3 samples from children with dual infections. In 2 mixed G1–G9 infections, G9, but not G1 RNA, was recovered, and G1 RNA was recovered from dipstick RNA in addition to G9 from a G9-infected child. A fourth dipstick from a G3 sample yielded a G3 and an equivocal G1 that did not appear in subsequent replicate heminested amplifications. In other words, 21 (92%) of 23 genotypes identified from RNA extracted from 18 fecal suspensions were also identified from RNA extracted from dipsticks, including 8 of 10 genotypes from 5 samples simultaneously infected with rotaviruses of 2 different genotypes.

Rotaviral VP7 RNA suitable for genotyping was also recovered from all 5 air-dried rotavirus-positive dipsticks that had been stored at room temperature for 5 years. The G genotypes of the rotavirus in the fecal suspensions from the 5 archived dipsticks from 2002 were unknown, and the suspensions were no longer available for comparison. One of the dipsticks was manufactured by Hy Laboratories; the remaining 4 were manufactured by Novamed. Initial amplification with generic G-type primers yielded generic G genotype amplification products of expected size (≈1,062 nt) upon gel electrophoresis. Three of the dipsticks yielded G3 RNA; the other 2 yielded G1 RNA. As proof of concept that P genotypes could be determined in addition to G genotypes, the P genotypes of RNA recovered from 2 of the 5-year-old rotavirus dipsticks with G3 RNA were also determined by direct sequencing of the RT-PCR products by using the generic P-type primers. Both were P[8] by sequence analysis (Table A1).

The G genotypes of 3 G1s, 2 G2s, 1 G3, and 1 G9 RNA extracted from dipsticks were confirmed by sequencing their heminested amplification products. Moreover, the sequences of the RNA extracted from each of the 7 dipsticks were identical to the sequence of the RNA extracted directly from the corresponding stool suspension. The lineages and sublineages of these and other isolates from the 2005–06 season were inferred from phylogenetic comparisons to equivalent segments of the 11 lineages of G1, the 2 lineages of G2, the 4 lineages of G3, and the 6 lineages of G9 described by Phan et al. (*15*), Page and Steele (*16*), Wang et al. (*17*), and Martinez-Laso et al. (*18*), respectively. The G3 phylogenetic tree also includes 3 sequences obtained from the 5-year-old dipsticks and 4 G3 isolates from among those with highest homology to G3 isolates from Israel identified in BLAST searches (http://blast.ncbi.nlm.nih.gov/Blast.cgi) (*19*). Specifically, all VP7 sequences for a given G genotype were aligned and truncated to the longest common segment. Four separate phylogenetic trees (Figure) were constructed because of the size difference between G genotype–specific heminested amplification products. The lineage or sublineage of each isolate from Israel (Table A) was inferred from the relative difference of each sequence compared with equivalent segments of previously determined lineages. For each genotype, a new lineage or sublineage was suggested when the sequence from Israel failed to closely group with the equivalent segment of >1 reference strains and the nucleotide differences between the strain from Israel and the reference strains were similar or greater than the differences among reference lineages and sublineages. The existence of such new lineages and sublineages would need to be confirmed with longer full-length open reading frame sequences. Finally, sequences from RNA recovered from dipsticks and/or from saline suspensions were used in BLAST searches to identify the most similar contemporary sequences. G1 isolates were most similar to isolates from Europe, the Far East, and South America; G2 isolates to isolates from the Far East; and G3 isolates to isolates from Europe and the Far East (Figure). G9 isolates were most similar to isolates from Africa, Europe, and the Far East.

## Discussion

The G1P[8] genotype predominated in central Israel during the 2005–06 rotavirus season as it did throughout Israel during 1991–1994 (*20*) and in northern Israel during 2007–2009 (*2**,**10*). However, the relative distributions of other genotypes differed. For example, G4P[8], absent during 2005–2008, was present in 32.3% of samples a decade earlier and reappeared in 2008–09 (*2*). Conversely, G9 genotypes, absent a decade earlier, were present in 15.7% of the 2005–06 isolates and 9.3% of the 2006–07 isolates. This substantial prevalence of G9 mirrors the global emergence of G9 among hospitalized children in the mid-1990s (*21**,**22*). In addition, double infections indicated by >1 G or P genotype rose from <2% during 1991–1994 (*20*) to 16.4% for the isolates in this study and 27% during 2007–08 (*2*). As indicated by Muhsen et al (*2*), evidence is good for rotavirus reassortants emerging in vivo from appropriate double infections. Phylogenetic analysis in the present study suggested that some G2 and G9 rotaviruses from Israel belonged to new sublineages or lineages, respectively.

In conclusion, air-dried affinity-concentrated virus from rotavirus-positive bands of dipsticks yielded RNA suitable for G genotyping and sequencing, even for dipsticks stored at room temperature for 5 years. Even though P genotyping and sequencing was performed only on RNA isolated from 2 of the 5-year old dipsticks, it is reasonable to assume that P genotyping and sequencing would have been possible for the rest based on the equivalent quality of the RNA extracted from the dipsticks and RNA extracted from stool suspensions. Thus, dipsticks can be used for recovery of molecular epidemiologic data by reference laboratories from diagnostic tests routinely performed in many clinical laboratories and point-of-care facilities. RNA extracted from these archived dipsticks will enable centralized laboratories to easily recover epidemiologically useful data from the samples of other laboratories and from regions lacking adequate laboratory facilities. In addition, centralized laboratories will be able to assess whether the introduction of universal rotavirus vaccination changed the distribution of rotavirus genotypes associated with severe rotavirus-associated acute gastroenteritis.

## Appendix

**Table A1:** Rotavirus genotypes and reference isolates, Israel

Genotype and lineage		Sublineage		Isolate name*		GenBank accession no.	Reference
G1 genotype 515 nt (equivalent to nt 479–nt 993 of isolate VN-281 (DQ508167)							
	1	a		Dhak8		AY631049	(15)
	1	b		VN-281		DQ508167	(15)
	1	c		Mvd9816		AF480293	(15)
	1	d		6916/JP		EF079067	(15)
	1	e		CH631		AF183837	(15)
	2	a		DC03		AF183859	(15)
	2	b		PA5/03		DQ377596	(15)
	2	c		JP-7026		EF079065	(15)
	2	d		PA164		DQ377588	(15)
	2	e		Russia-407		S83903	(15)
	3	b		Israel-56		U26376	(15)
	4	None		K54		U26377	(15)
	5	None		PA5/90		QD377573	(15)
	6	None		AU19		AB018697	(15)
	7	None		Ban-56		U26366	(15)
	8	None		Egypt-7		U26373	(15)
	9	A		P19		DQ377593	(15)
	10	None		SW2021		AF426162	(15)
	11	b		C60		L24164	(15)
	2	c		RoV_09_ISR05[G1.2c]D		FN582119	New
	2	c		RoV_09_ISR05[G1.2c]S		FN298858	New
	1	a		RoV_10_ISR05[G1.1a]D		FN582120	New
	1	a		RoV_10_ISR05[G1.1a]S		FN582121	New
	2	c		RoV_14_ISR05[G1.2c]S		FN582122	New
	1	a		RoV_41_ISR05[G1.1a]D		FN582123	New
	1	a		RoV_41_ISR05[G1.1a]S		FN298860	New
G2 genotype 547 nt (equivalent to nt 455–nt 1,001 of isolate TN1529 (AY261357)							
			1	None	sa514 gr	ay261338	(16)
			2	a	KY3303	AY261350	(16)
			2	b	SA3958GR	AY261344	(16)
			2	c	TN1529	AY261357	(16)
			2	d	BF3767	AY261355	(16)
			2	e†	RoV_39_ISR05[G2.2e]D	FN582124	New
			2	e†	RoV_39_ISR05[G2.2e]S	FN298861	New
			2	B	RoV_51_ISR05[G2.2b]S	FN298862	New
			2	e†	RoV_63_ISR06[G2.2e]D	FN298864	New
			2	e†	RoV_63_ISR06[G2.2e]S	FN298863	New
G3 genotype 274 nt (equivalent to nt 701–nt 982 of isolate AU-1 (D86271)							
			1	None	CMH222	AY707792	(17)
			2	None	CHW17	D86276	(17)
			2	None	CH-55	D86274	(17)
			3	1	AU-1	D86271	(17)
			3	2	TK28	D86283	(17)
			3	3	CMH120/04	DQ923797	(17)
			3	4	E887	EU708579	(17)
			3	4	CMH272	AY707790	GenBank
			3	4	Rus32	EF495127	GenBank
			3	4	47–2536Madrid	DQ440624	GenBank
			3	4	VN-933	EF495123	GenBank
			4	None	CMP096 (porcine)	DQ256502	(17)
			3	4	RoV_28_ISR05[G3.3.4]D	FN298865	New
			3	4	RoV_28_ISR05[G3.3.4]S	FN298866	New
			3	4	RoV_64_ISR02[G3.3.4]D‡	FN298867	New
			3, NA¶	4, NA	RoV_65_ISR02[G3.3.4]D,‡ RoV_65_ISR02[P[8]]D§	FN298868, HQ174462	New
			3, NA	4, NA	RoV_68_ISR02[G3.3.4]D,‡ RoV_68_ISR02[P[8]]D§	FN298869, HQ174463	New
G9 genotype 207 nt (equivalent to nt 77–nt 983 of isolate AU32 (AB045372**)**							
			1	None.	AU32	AB045372	(18)
			2	None	116E	L14072	(18)
			3	None	INL1	AJ250277	(18)
			4	None	97'SZ37	AF260959	(18)
			5	None	OM67	AJ491179	(18)
			6	None	SP1542	AB091753	(18)
			7†	None	RoV_24_ISR05[G9.7]S	FN298870	New
			8†	None	RoV_56_ISR06[G9.8]D	FN298873	New
			8†	None	RoV_56_ISR06[G9.8]S	FN298872	New
			8†	None	RoV_57_ISR06[G9.8]S	FN298874	New
			8†	None	RoV_60_ISR06[G9.8]S	FN298875	New

*Isolate names were taken from European Molecular Biology Laboratory/GenBank/DNA Data Bank of Japan. Israeli samples have been named RoV_the isolate number_ISR for Israel and a 2-digit code indicating the year the sample was sent to the laboratory for initial testing. †New lineages or sublineages inferred by phylogenetic comparison to equivalent segments from reference lineages and sublineages, as described in the text. ‡G genotypes determined from RNA isolated from dipsticks stored at room temperature for 5 y. RNA from all other dipsticks was extracted 1–6 wk after the rapid test strips (dipsticks) were used. §P genotypes determined from RNA isolated from dipsticks stored at room temperature for 5 y. The 457-nt sequence is equivalent to nt 222– 678 of a G3P[8] isolate, 95–87 (AB008290). ¶NA, not applicable.

## References

[1] Parashar UD, Hummelman EG, Bresee JS, Miller MA, Glass RI. Global illness and deaths caused by rotavirus disease in children. Emerg Infect Dis. 2003;9:565–72. 12737740 10.3201/eid0905.020562 PMC2972763

[2] Muhsen K, Shulman L, Rubinstein U, Kasem E, Kremer A, Goren S, Incidence, characteristics, and economic burden of rotavirus gastroenteritis associated with hospitalization of Israeli children <5 years of age, 2007–2008. J Infect Dis. 2009;200(Suppl 1):S254–63. 10.1086/605425 19817606

[3] Widdowson MA, Bresee JS, Gentsch JR, Glass RI. Rotavirus disease and its prevention. Curr Opin Gastroenterol. 2005;21:26–31. 15687881

[4] Parashar UD, Gibson CJ, Bresse JS, Glass RI. Rotavirus and severe childhood diarrhea. Emerg Infect Dis. 2006;12:304–6. 16494759 10.3201/eid1202.050006 PMC3373114

[5] Parashar UD, Burton A, Lanata C, Boschi-Pinto C, Shibuya K, Steele D, Global mortality associated with rotavirus disease among children in 2004. J Infect Dis. 2009;200(Suppl 1):S9–15. 10.1086/605025 19817620

[6] Estes MK, Cohen J. Rotavirus gene structure and function. Microbiol Rev. 1989;53:410–49. 2556635 10.1128/mr.53.4.410-449.1989 PMC372748

[7] Estes MK, Kapikian AZ. Rotaviruses. In: Knipe DM, Howley PM, Griffin DE, Lamb RA, Martin MA, Roizman B, et al., editors. Fields virology. 5th ed. Philadelphia: Lippincott Williams & Wilkins; 2007. p. 1917–74.

[8] Vesikari T, Matson DO, Dennehy P, Van Damme P, Santosham M, Rodriguez Z, Safety and efficacy of a pentavalent human–bovine (WC3) reassortant rotavirus vaccine. N Engl J Med. 2006;354:23–33. 10.1056/NEJMoa052664 16394299

[9] Ruiz-Palacios GM, Perez-Schael I, Velazquez FR, Abate H, Breuer T, Clemens SC, Safety and efficacy of an attenuated vaccine against severe rotavirus gastroenteritis. N Engl J Med. 2006;354:11–22. 10.1056/NEJMoa052434 16394298

[10] Muhsen K, Shulman L, Kasem E, Rubinstein U, Shachter J, Kremer A, Effectiveness of rotavirus vaccines for prevention of rotavirus gastroenteritis–associated hospitalizations in Israel: a case–control study. [Epub ahead of print.]. Hum Vaccin. 2010;6. 20448471 10.4161/hv.6.6.11759

[11] Gouvea V, Glass RI, Woods P, Taniguchi K, Clark HF, Forrester B, Polymerase chain reaction amplification and typing of rotavirus nucleic acid from stool specimens. J Clin Microbiol. 1990;28:276–82. 2155916 10.1128/jcm.28.2.276-282.1990 PMC269590

[12] Gunasena S, Nakagomi O, Isegawa Y, Kaga E, Nakagomi T, Steele AD, Relative frequency of VP4 gene alleles among human rotaviruses recovered over a 10-year period (1982–1991) from Japanese children with diarrhea. J Clin Microbiol. 1993;31:2195–7. 8396591 10.1128/jcm.31.8.2195-2197.1993 PMC265721

[13] Thompson JD, Gibson TJ, Plewniak F, Jeanmougin F, Higgins DG. The CLUSTAL_X windows interface: flexible strategies for multiple sequence alignment aided by quality analysis tools. Nucleic Acids Res. 1997;25:4876–82. 10.1093/nar/25.24.4876 9396791 PMC147148

[14] Perrière G, Gouy M. WWW-query: an on-line retrieval system for biological sequence banks. Biochimie. 1996;78:364–9. 10.1016/0300-9084(96)84768-7 8905155

[15] Phan TG, Khamrin P, Quang TD, Dey SK, Takanashi S, Okitsu S, Detection and genetic characterization of group A rotavirus strains circulating among children with acute gastroenteritis in Japan. J Virol. 2007;81:4645–53. 10.1128/JVI.02342-06 17301134 PMC1900178

[16] Page NA, Steele AD. Antigenic and genetic characterization of serotype G2 human rotavirus strains from the African continent. J Clin Microbiol. 2004;42:595–600. 10.1128/JCM.42.2.595-600.2004 14766822 PMC344437

[17] Wang YH, Kobayashi N, Zhou X, Nagashima S, Zhu ZR, Peng JS, Phylogenetic analysis of rotaviruses with predominant G3 and emerging G9 genotypes from adults and children in Wuhan, China. J Med Virol. 2009;81:382–9. 10.1002/jmv.21387 19107964

[18] Martinez-Laso J, Roman A, Head J, Cervera I, Rodriguez M, Rodriguez-Avial I, Phylogeny of G9 rotavirus genotype: a possible explanation of its origin and evolution. J Clin Virol. 2009;44:52–7. 10.1016/j.jcv.2008.08.022 18977689

[19] Zhang Z, Schwartz S, Wagner L, Miller W. A greedy algorithm for aligning DNA sequences. J Comput Biol. 2000;7:203–14. 10.1089/10665270050081478 10890397

[20] Silberstein I, Shulman LM, Mendelson E, Shif I. Distribution of both rotavirus VP4 genotypes and VP7 serotypes among hospitalized and nonhospitalized Israeli children. J Clin Microbiol. 1995;33:1421–2. 7615771 10.1128/jcm.33.5.1421-1422.1995 PMC228184

[21] Santos N, Hoshino Y. Global distribution of rotavirus serotypes/genotypes and its implication for the development and implementation of an effective rotavirus vaccine. Rev Med Virol. 2005;15:29–56. 10.1002/rmv.448 15484186

[22] Santos N, Volotao EM, Soares CC, Campos GS, Sardi SI, Hoshino Y. Predominance of rotavirus genotype G9 during the 1999, 2000, and 2002 seasons among hospitalized children in the city of Salvador, Bahia, Brazil: implications for future vaccine strategies. J Clin Microbiol. 2005;43:4064–9. 10.1128/JCM.43.8.4064-4069.2005 16081952 PMC1233902

